# The complete mitochondrial genome of the Aral barbel *Luciobarbus brachycephalus* (Cypriniformes: Cyprinidae: Barbinae)

**DOI:** 10.1080/23802359.2019.1678439

**Published:** 2019-10-21

**Authors:** Haifeng Jiang, Longwu Geng, Jian Yang, Guangxiang Tong, Chenyu Li, Wei Xu

**Affiliations:** National and Local United Engineering Laboratory for Freshwater Fish Breeding, Heilongjiang River Fisheries Research Institute, Chinese Academy of Fishery Sciences, Harbin, China

**Keywords:** *Luciobarbus brachycephalus*, mitogenomics, barbinae

## Abstract

The Aral barbel (*Luciobarbus brachycephalus*) is an important commercial species in Aral sea basin and its population is decreasing rapidly. However, the genetic resource available for this species as well as other barbels in barbinae is limited. In this study, we described the complete mitochondrial genome of *L. brachycephalus*. The mitochondrial genome is 16,603 bp in length containing 13 protein-coding genes (PCGs), two ribosomal RNAs, 22 tRNAs and a control region. The overall base composition of *L. brachycephalus* mtDNA is: 31.2% for A, 27.8% for C, 16.5% for G, 24.4% for T.

The Aral barbel (*Luciobarbus brachycephalus*) is a valuable species belongs to genus *Luciobarbus* within family Cyprinidae Barbinae, occurs in the Aral basin, Chu drainage and southern and western Caspian Sea (Coad 1998). However, its total population has declined by at least 30% in the past 30 years and currently listed on the IUCN Red List as Vulnerable (VU) species (Esmaeili et al. 2017). At present, *L. brachycephalus* is an important economic species in China since it was introduced in 2003. In this study, we determined complete mitochondrial DNA sequence of the *L. brachycephalus* and explored the phylogenetic relationship within barbinae.

Adult specimen was obtained from Hulan aquaculture experimental station in Harbin, Heilongjiang province, China (45.75° latitude, 126.34° longitude) and is deposited in the Museum of Heilongjiang River Fisheries Research Institute with identifier HLJ190901. The morphological characters that can diagnose *L. brachycephalus* from other species of Luciobarbus were according to previous study (Kottelat and Freyhof 2007). The total DNA was extracted from fin tissue using the phenol–chloroform method (Barnett and Larson 2012), and the complete mitogenome was amplified with 16 pairs of primers. Overlapping fragments obtained by sequencing were edited and aligned using BioEdit 7.0.9.0 (Hall 1999). The calculation of base composition and phylogenetic analysis was conducted by MEGA6.0 software (Tamura et al. 2013).

The final assembly 16,603 bp of the mitogenome of the *L. brachycephalus* (GenBank accession numbers MN460368) contained 13 PCGs, two ribosomal RNA genes, 22 tRNAs and a putative Dloop with a nucleotide composition of A% = 31.2, G%= 16.5, T% = 24.4 and C% = 27.8. The genome has an overall AT content of 55.6% and GC content of 44.4%. Eight tRNA genes (Ala, Asn, Cys, Gln, Glu, Pro, Tyr, Ser) and one protein-coding gene (ND6) were encoded on light strand with the others on heavy strand. Twelve of 13 PCGs started with the typical codon ATG, whereas COI began with GTG. Nine PCGs had complete termination codons (TAA for ND1, COI, ATP8, ATP6, COIII, ND4L, ND5, ND6 and TAG for ND2). The remaining four genes ended with the incomplete termination codon T (COII, ND3, ND4 and Cyt b). The 12S rRNA and 16S rRNA are 953 and 1640 bp respectively, which are located in the typical positions between tRNA-Phe and tRNA-Leu, separated by tRNA-Val. The intergenic spacer sequences ranged from 1 to 32 bp and the largest spacer located in a cluster of five tRNA genes (WANCY region) between tRNA-Asn and tRNA-Cys genes. The Dloop located between tRNA-Pro and tRNA-Phe, was846 bp in length.

To explore the phylogenetic position of this Aral barbel, a phylogenetic tree was constructed based on the neighbor-joining (NJ) analysis of other determined barbel mitogenomes and other closely related genus in Cyprinidae (Figure 1). The phylogenetic analysis shows that *L. brachycephalus* is closely related to *L.sclateri*, and genus Luciobarbus has closest relationship with genus Capoeta, which is in accord with previous study (Ghanavi et al. 2016).

## Figures and Tables

**Figure 1.**
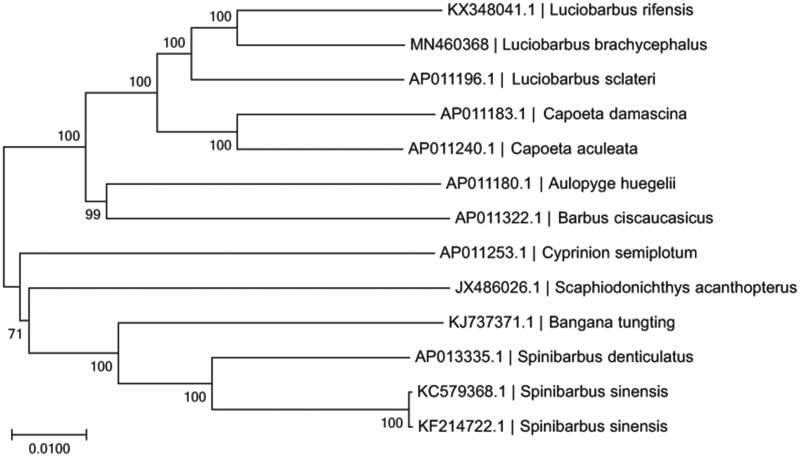
Phylogennetic analysis of *L. brachycephalus* based on the entire mtDNA genome sequences of 13 cyprinidae fish available in GenBank. Numbers above the nodes indicate 1000 bootstrap values. Accession numbers are shown before species names.
